# A reproducible web-scraping pipeline for collecting geolocated German online job advertisements at scale

**DOI:** 10.1016/j.mex.2026.104166

**Published:** 2026-09-14

**Authors:** Matthias Kapa, Kerstin J. Schaefer

**Affiliations:** a Leibniz University Hannover, Institute of Economic and Cultural Geography, Schneiderberg 50, Hannover, 30167, Germany; b Utrecht University, Human Geography and Spatial Planning, Princetonlaan 8a, Utrecht, 3584 CB, the Netherlands

**Keywords:** Web scraping, Online job advertisements, Scrapy, Python, Labour market data, Data collection pipeline, Privacy anonymisation, Germany

## Abstract

Online job advertisements (OJAs) provide near-real-time data on labour market skill demand including location information but are predominantly accessed through costly commercial providers whose collection and processing methods are not publicly documented. We present a fully reproducible, open-source web-scraping pipeline that systematically collects OJAs from StepStone, one of Germany's largest job portals. The pipeline combines the Scrapy crawling framework with Playwright-based headless browser automation to handle dynamic JavaScript-rendered content, implements deduplication logic, and deploys a locally run large language model (AI4Privacy) to censor personally identifiable information in compliance with the General Data Protection Regulation. Applied from April to July 2023, the pipeline collected 342,452 unique OJAs. Validation against the commercial provider Lightcast confirms that, for single-portal scrapes, regional coverage (79% vs 80% of German municipalities) and spatial distribution are broadly comparable to a commercial provider, though volume and cross-portal reach differ. However, because of the smaller overall size of the single-portal dataset, there might be drawbacks when it comes to the analysis of smaller sectors. The anonymised dataset and cleaning scripts are released in machine-readable JSON format on Mendeley Data.

• A Scrapy + Playwright pipeline reliably collects large-scale OJAs from a dynamic, JavaScript-rendered job portal.
• A locally deployed LLM (AI4Privacy) censors personally identifiable data in job ad text before release.
• Validation against Lightcast shows that the single-portal scrape reproduces the aggregate NUTS-2 regional structure of the German OJA landscape (Spearman ρ = 0.91), though direct record-level URL overlap is low owing to cross-source deduplication and URL instability.

## Graphical abstract

**Figure:**
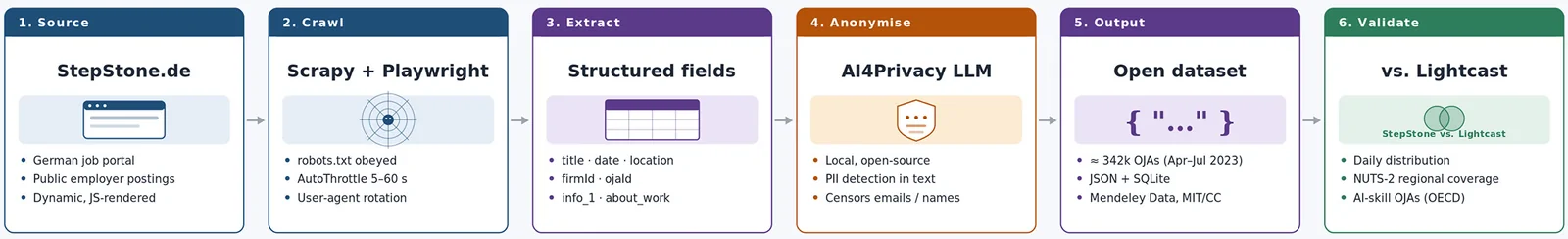

**Specifications table**

**Table:** 

**Subject area**	
**More specific subject area**	Labour Market Research; Computational Social Science; Applied Data Collection
**Name of your method**	Online Job Advertisement Web-Scraping Pipeline (OJA-Scraper)
**Name and reference of original method**	Scrapy web-crawling framework: Scrapy (2025). Scrapy 2.13 documentation. Playwright browser automation: Playwright (2024). scrapy-playwright: A Python framework integrating Playwright with Scrapy. https://github.com/scrapy-plugins/scrapy-playwright. Version 0.4.0.
**Resource availability**	Dataset (Mendeley Data, JSON): https://doi.org/10.17632/wnt24rfrwz.2 Scraping code: https://github.com/maciek85k/scraper Censor code: https://github.com/maciek85k/stepstoneCensor

## Background

Official statistics compiled by the German Federal Employment Agency (Bundesagentur für Arbeit) do not capture the full range of open vacancies because reporting is voluntary, creating a persistent gap in labour market monitoring. Online job advertisements (OJAs) posted on commercial job portals have emerged as a near-real-time alternative, offering rich textual information on skill requirements, occupation descriptions, and workplace location. However, most researchers relying on OJA data purchase access to commercial aggregators, such as Lightcast (formerly Burning Glass) or Textkernel, whose crawling schedules, deduplication rules, and pre-processing pipelines are not fully disclosed. Efforts to build a shared, harmonised OJA infrastructure at the European level are ongoing but have not yet produced a freely available German-language corpus [1]. This opacity limits reproducibility and imposes a financial burden on research projects with limited budgets [2,3].

Web scraping offers a transparent and cost-effective alternative: data are collected directly from the source portal, every processing step is documented and version-controlled, and the resulting dataset can be shared openly. Advances in open-source crawling frameworks, headless browser automation, and cloud computing have made large-scale scraping technically feasible for academic teams [[4], [5], [6]]. At the same time, scraping introduces its own methodological challenges: single-portal coverage may miss certain labour market segments, JavaScript-rendered content requires more sophisticated tooling than simple HTML parsing, and publicly accessible postings may inadvertently contain personally identifiable information (PII) that must be handled in compliance with the General Data Protection Regulation (GDPR) [7].

This article describes a reusable scraping pipeline designed to address these challenges. While web-mining approaches for the German labour market have recently emerged [8], they typically focus on combining multiple structured sources using shared classification schemes rather than providing a standalone, reproducible scraping pipeline for a single major portal. The pipeline was originally developed to study the spatial distribution of AI-skill demand in Germany, which is a niche subset within the overall OJA data. We therefore validate not only the overall coverage but also use this subset to test how smaller samples are represented. While the dataset is well suited for OJA-based labour market analysis of white-collar and knowledge-intensive occupations in Germany [9], our approach, while having size limitations against larger commercial providers, proves to be more transparent and reproducible. Moreover, the pipeline is adaptable to other portals with similar architecture.

## Method details

### Legal & ethical framework

StepStone's robots.txt file (https://www.stepstone.de/robots.txt) is the standard, protocol-based mechanism by which a website tells automated agents which resources should not be accessed. It consists of simple directives such as User-Agent, Disallow, Allow, and, optionally, Crawl-delay that specify the URL-path prefixes that are off-limits and the minimum pause between requests. Although adherence to robots.txt is not a legal requirement, it is widely regarded as the ethical baseline for web scraping under most jurisdictions and is often incorporated into a site's Terms of Service [10]. Ignoring it can be interpreted as a breach of good-faith conduct and, in some jurisdictions, may be used as evidence of intentional circumvention of access controls [11]. Respecting these directives yields multiple benefits: it reduces server load by preventing needless requests to high-traffic or dynamically generated pages; it lowers the risk of IP-address blocking, since many sites automatically blacklist agents that repeatedly violate their robots.txt; and it yields cleaner, more relevant data because the pages marked as off-limits are typically low-value, saving storage space and downstream cleaning effort.

In practice, we first fetch and then check each candidate URL with the fetch method using our crawler's user-agent string, skipping disallowed URLs and throttling request rates when a Crawl-delay is specified. By treating robots.txt as a binding ethical contract, and where applicable, a contractual term of StepStone's Terms of Service, we can ensure operational interruptions and unnecessary data noise are minimised while honouring the publisher's intent to keep certain resources private. We even contacted StepStone multiple times to inform them of our project and request permission to scrape their data, but received no response.

### Scraping architecture

To efficiently handle the challenges of web scraping, we utilised Scrapy [12], a Python framework designed for extracting data from websites. Scrapy excels in sending HTTP requests and parsing HTML responses, making it particularly effective for dealing with static content. However, for dynamic content generated through JavaScript, additional tools are necessary.

Scrapy crawls websites by following hyperlinks while automatically respecting robots.txt directives and throttling request rates to avoid overloading target servers. For our project, we configure the spider to collect only URLs that contain the term "Stellenanzeige" ("job advertisement"), thereby restricting the crawl to genuine job-posting pages on StepStone. Scrapy's built-in exporters then write the extracted data to JSON format, making it well-suited for large-scale data-collection pipelines [12].

To enhance Scrapy's capabilities, we integrated it with Playwright [13], a platform that facilitates browser automation. This integration enables Scrapy to simulate real user interactions and efficiently render JavaScript-heavy pages. By combining these tools, we developed a comprehensive workflow that enhances efficiency and reduces the likelihood of triggering automated rate-limiting or blocking measures, thereby improving collection stability and completeness. This setup is particularly advantageous for large-scale projects that involve dynamic content and complex interactions, ensuring a smooth and efficient data extraction process.

StepStone's UI also includes a "related-jobs" widget, which suggests additional job-posting URLs that are automatically enqueued by the spider. These suggestions, based on initial search terms, include potential new hyperlinks, allowing Scrapy to navigate and scrape any novel links it encounters. As long as StepStone continues to suggest fresh OJAs, the crawling process remains active.

The extraction itself proceeds in two steps. First, a crawling rule follows only hyperlinks that match the URL pattern of individual job postings (stellenangebote...html) and are located in the result list. Second, on each posting page, the spider fills a structured item with eight fields using CSS selectors: the page title, the posting date, the date of scraping, the employer name and the link to its StepStone profile (firmId), the posting URL itself (ojaId), the job description (about_work), and the further details block (info_1). The URL-based fields also serve as identifiers during deduplication (see Deduplication). Records in which the selectors return no content beyond the mandatory fields are discarded at the item pipeline stage.

A practical weakness of this extraction approach is the fragility of the CSS selectors. The spider identifies links and data fields through StepStone’s class names (for example, a.resultlist-10m88ja for links to individual postings). These class names are generated by the site’s styling framework and can change with any front-end release, even when the page content itself stays the same. A broken selector does not cause a crash but silently yields empty fields. The mandatory-field check in the item pipeline (see Data Description) therefore doubles as a detection mechanism: a sudden spike in discarded observations indicates that the selectors need updating. More robust alternatives, such as parsing the structured JSON-LD data embedded in the posting pages, would reduce this maintenance burden and are a natural extension of the pipeline.

### Configuration details

The OJA crawling system is built on Scrapy, a widely-used Python framework for large-scale web extraction, and is extended with Playwright to handle modern, JavaScript-driven job-listing portals. The configuration adopts a "responsible-by-design" philosophy: it respects site owners' policies, mimics genuine browsers, and adapts its request rate to the observed responsiveness of each target server.

The crawler advertises itself under the name “jobs” and is organised so that all spider definitions reside in the jobs.spiders package. By enabling ROBOTSTXT_OBEY = True, the system automatically parses each site's robots.txt file and refrains from accessing URLs that are disallowed, thereby ensuring compliance with the explicit crawling directives published by the host.

To retrieve content that is generated client-side, the download pipeline delegates every HTTP and HTTPS request to Playwright's download handler. This delegation is expressed through the DOWNLOAD_HANDLERS mapping, which routes both protocols to Scrapy Playwright Download Handler. Because Playwright operates on Python's asyncio event loop, the underlying Twisted reactor is switched to Asyncio Selector Reactor, allowing Scrapy's asynchronous engine to cooperate seamlessly with Playwright's asynchronous page lifecycle.

Network instability and transient server errors are mitigated by a custom retry strategy. The native Scrapy Retry Middleware is disabled and replaced with a project-specific implementation (jobs.retry.Retry Middleware) executed with a priority of 550 in the downloader-middleware chain. The maximum number of retry attempts is raised to ten (RETRY_TIMES = 10), and the set of exceptions that trigger a retry is extended to include Playwright's Timeout Error. Consequently, the crawler makes multiple, controlled attempts to fetch a page before abandoning it, increasing the overall recall of the extraction process.

To reduce the likelihood of being flagged as a threat, each outgoing request is assigned a randomly selected user-agent string from a curated list of contemporary desktop browsers (e.g., Chrome 111 on Windows and macOS). The Rotate User Agent Middleware performs this rotation, while cookies are globally disabled (COOKIES_ENABLED = False) because the majority of job-listing pages do not require session state. Redirect handling remains enabled (REDIRECT_ENABLED = True) so that legitimate URL changes are followed automatically.

The crawler's request rate is governed by Scrapy's AutoThrottle extension. With AUTOTHROTTLE_ENABLED = True, the system begins each crawl with a five-second pause between requests (AUTOTHROTTLE_START_DELAY = 5). If the target server exhibits high latency, the delay can expand up to sixty seconds (AUTOTHROTTLE_MAX_DELAY = 60). The algorithm targets an average concurrency of one request per remote host (AUTOTHROTTLE_TARGET_CONCURRENCY = 1.0), thereby avoiding excessive load and lowering the risk of IP bans.

### Crawl statistics

Scrapy provides a framework that orchestrates every step of a crawl. We configure the logger to use the INFO level, which yields a concise overview of each spider's activity without overwhelming the log file (scrapy.log). Table 1 presents a summary of the logger's statistics.

**Table 1:** Scrapy crawl statistics for ten consecutive crawls (April–July 2023). crawl 3 (15 April) shows elevated 403 responses due to a temporary IP-based block.

Crawl	Start date	Finish date	HTTP req.	200 OK	301	403	410	Duplicates	Max depth	Finish
1	2023-04-04	2023-04-05	100,673	98,772	979	355	522	863,156	1808	finished
2	2023-04-11	2023-04-12	94,134	92,867	990	—	234	834,748	1801	finished
3	2023-04-15	2023-04-16	85,060	44,009	448	40,566	32	355,310	1814	finished
4	2023-04-17	2023-04-18	93,935	92,293	1047	—	567	829,838	1742	finished
5	2023-04-21	2023-04-23	100,882	96,904	953	—	2992	866,131	1883	finished
6	2023-05-03	2023-05-04	95,644	92,868	1053	—	1706	830,927	1759	finished
7	2023-06-06	2023-06-12	101,076	94,524	1444	—	5098	844,064	1926	finished
8	2023-06-26	2023-06-26	101,042	93,539	1674	—	5813	832,196	1839	finished
9	2023-07-05	2023-07-11	99,383	92,699	1754	—	4918	827,452	1832	finished
10	2023-07-17	2023-07-23	101,379	94,726	1293	—	5339	844,957	1880	finished

Between 4 April 2023 and 23 July 2023, we conducted ten consecutive Scrapy crawls of the target website. Each crawl lasted between one and seven days. The overall number of HTTP requests remained stable; per crawl, it hovered around 100,000 (ranging from 85,000 to 101,000). The distribution of response status codes revealed that the vast majority of requests succeeded, with approximately 95% returning a 200 OK status code.

The only notable deviation occurred in the third crawl (15 April 2023), where successful responses (HTTP 200) dropped to 44,009 (approximately 44% of all responses), while access-denied responses (HTTP 403 Forbidden) surged to 40,566. This pattern suggests that the crawler was temporarily blocked by the server, likely due to a rate limit or an IP-based ban that was lifted before the next run.

The effect of this block on the final dataset was limited, although we cannot rule out that some postings were missed. The 40,566 blocked requests are an upper bound on the pages missed in Crawl 3, and this figure also includes result-list and navigation pages, not only job postings. In addition, each crawl session started anew and did not carry over a list of visited URLs, so pages blocked in Crawl 3 were simply requested again in later sessions. Crawl 4 started only two days later (17 April) and ran normally again (92,293 successful responses), meaning that postings blocked in Crawl 3 that were still online could be collected in Crawl 4 or a later session. A permanent loss is therefore only likely for postings that were blocked and taken offline within these two days. Assuming that a posting typically remains online for about one month, this concerns at most roughly 2800 postings, or well under 1% of the released dataset. Since we did not match the blocked URLs against later crawls, this figure is an estimate rather than an exact count.

Other error codes (301, 410, 502, 503, 504) were sparse and displayed no systematic trend across the ten runs. 301 (permanent redirects) remained roughly constant, averaging approximately 1000 per crawl. 410 (Gone) increased gradually from 522 in the first crawl to over 5000 in the final two crawls, reflecting the progressive removal of stale URLs from the site. Duplicate requests, those that Scrapy recognised as already visited, were abundant, averaging approximately 830,000 to 866,000 per crawl (∼84% of the total URL fingerprints seen by the scheduler, including both downloaded and pre-filtered requests), indicating that the spider's breadth-first strategy repeatedly encountered the same resources as the crawl depth increased. The maximum crawl depth ranged from 1,742 to 1926. All crawls terminated with the Scrapy-generated "finished" reason, confirming that the spider reached its predefined termination conditions. The single successful robots.txt fetch (status 200) in every run demonstrates that the spider respected the site's robots policy throughout the experiment.

### Deduplication

Duplicate records are handled at three distinct stages of the pipeline. Within a single crawl session, Scrapy’s built-in duplicate-request filter (RFPDupeFilter) computes a fingerprint for every enqueued request from the canonicalised URL: query parameters are sorted alphabetically, the URL fragment is removed, and percent-encodings as well as the character case of scheme and host are normalised (implemented via the w3lib library). Requests whose fingerprint has already been seen within the session are discarded before download. These filtered requests constitute the “Duplicates” column of Table 1 (approximately 830,000–866,000 per crawl, ∼84% of the total URL fingerprints seen by the scheduler, including both downloaded and pre-filtered requests) and arise chiefly because the related-jobs widget repeatedly suggests postings that the spider has already visited.

**Fig. 1:**
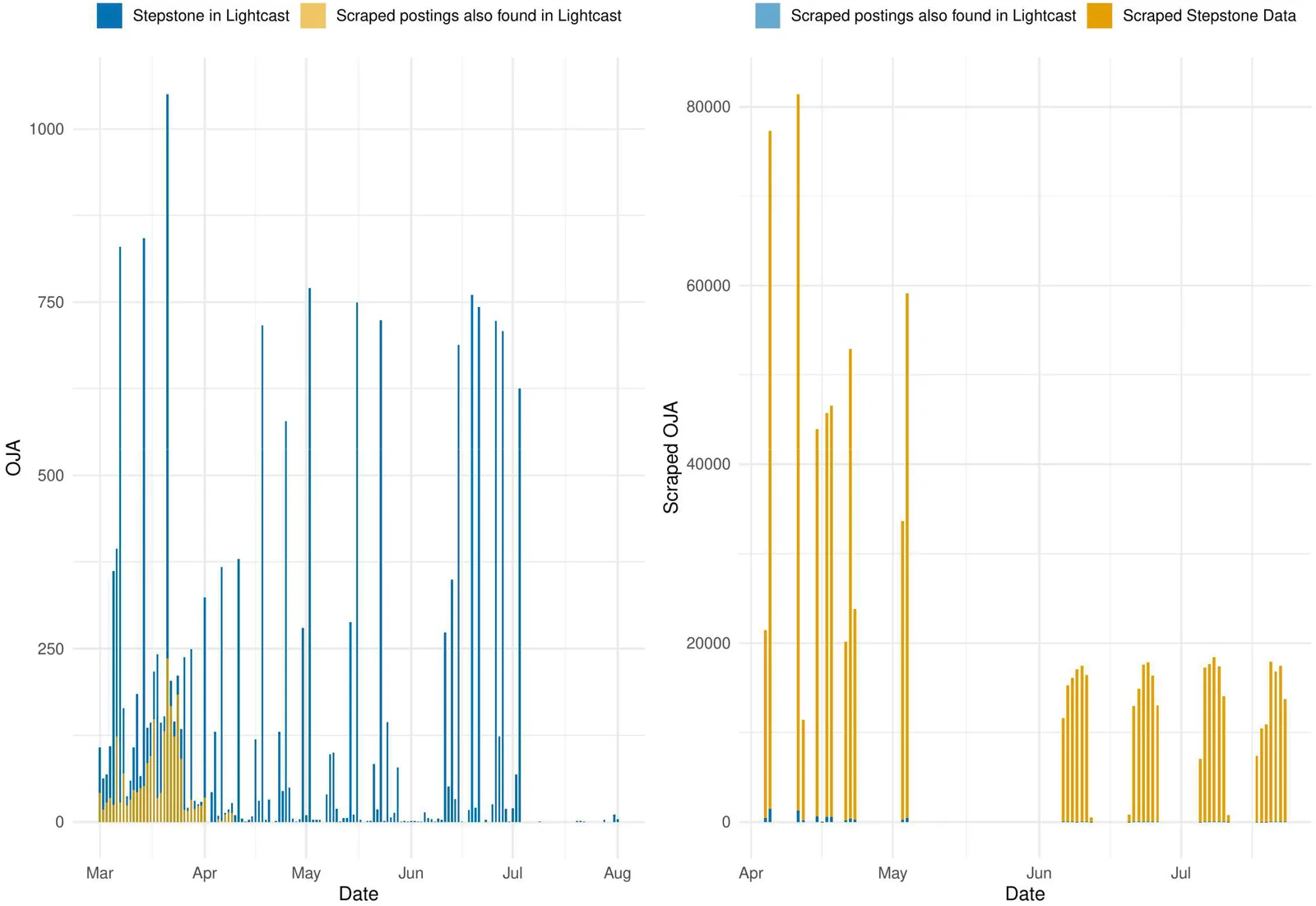
Daily distribution of collected OJAs.

The pipeline additionally employs the scrapy-deltafetch spider middleware (DELTAFETCH_ENABLED = True). DeltaFetch keeps a local database of pages that have already yielded a scraped item and skips them on later requests. In our setup, however, each of the ten crawl sessions started with a fresh state, so this mechanism prevented repeated downloads only within a session and did not track URLs between crawls. A posting that remained live on StepStone was therefore typically collected again in later sessions, which explains why the raw export contains repeated observations of the same vacancy (cf. the right panel of Fig. 1).

The final deduplication step is performed after collection, at the dataset level. The deduplication key is the ojaId field, i.e. the permanent URL of the individual OJA page (see Table 2), normalised in the same manner as described above. All raw records

**Table 2:** Variables extracted for each OJA. mandatory fields must be present for an observation to be retained in the dataset.

Variable	Description	Mandatory
title	Job headline	Yes
date_scrape	ISO 8601 timestamp recorded by the crawler at download time	No
firmId	Canonical URL of the employer's StepStone profile page; used as stable firm identifier	Yes
ojaId	Permanent URL of the individual OJA page; used as unique vacancy identifier	Yes
about_work	Tag-cloud block containing workplace location, scope, contract type, study/education mode, and posting age	No
info_1	Full free-text description, split into up to four sub-sections: firm overview, task/programme description, requirements, and benefits	No

### Data description

The data collected from StepStone comprises raw text data devoid of HTML from each OJA. Each observation includes the following variables: title, date_scrape, firmId, ojaId, info_1, and about_work (see Table 2 for details). Table 2 also indicates whether a variable is mandatory for an OJA to be included as an observation in the dataset. This information is important for enabling data quality checks. It is therefore possible for observations to contain only the mandatory variables, in case our scraper did not collect any further information. In total, 526 online OJAs lacked any further information beyond the mandatory fields and were discarded at the item pipeline stage.

The variable about_work contains information on the location of the workplace or the type of contract. The raw text in variable info_1 can be divided into four parts: information on the workplace, description of the task, requirements to get the position, and some benefits. While some firms omit benefits, others provide extremely concise descriptions.

We identified the language of each OJA with textcat an R package [14], applied to the info_1 field. We identified 331,024 advertisements as German and 10,899 as English. A small number of OJAs in French and Esperanto were also present (the Esperanto labels almost certainly reflect language-identification errors by the textcat package rather than genuine Esperanto postings).

Initially, our objective was to prevent the download of personal data by excluding the ability to scrape the contact field from StepStone. However, we discovered that some of the OJAs' content inadvertently contained contact information. To address this issue before data release, a locally deployed instance of the AI4Privacy multilingual anonymiser model [3] (llama-ai4privacy-multilingual-categorical-anonymiser-openpii) was applied to the info_1 and about_work fields. The Hugging Face platform offers a variety of pretrained models, and AI4Privacy [3] provides a model specifically trained to censor data for privacy concerns. The censor script is available at https://github.com/maciek85k/stepstoneCensor. The censoring step is implemented as an R script that calls the locally stored model through the reticulate interface. Postings are processed in batches; the model classifies each token, and detected PII spans are replaced with category placeholders such as [MASKED_GIVENNAME] or [MASKED_EMAIL], covering more than 20 categories including names, e-mail addresses, phone numbers, and street addresses. Each record additionally receives a flag indicating whether any PII was found, which allowed the affected postings to be reviewed separately. The whole procedure runs locally, so no posting text leaves the research machine. To assess the anonymisation quality, we inspected a stratified random sample of 200 postings (100 flagged by the model as containing PII, 100 unflagged) by comparing the original and the censored text side by side. The model redacted 78 of 79 personal data instances in the sample, including all 19 e-mail addresses and 33 telephone numbers of contact persons as well as 26 contact names; one contact person’s name remained unmasked while the associated telephone number was caught. The affected record was corrected in the released dataset (V2). In addition, 107 non-personal strings such as dates, job reference numbers, and postcodes were masked, spread across 74 of the 200 sampled postings (37%). This over-redaction removes some content but releases no personal data, so we consider this error direction acceptable for a privacy-preserving release.

### Software versions and environment

Python 3.10; Scrapy 2.13.2; scrapy-playwright 0.0.26; Playwright for Python 1.33; scrapy-proxy-pool; scrapy-deltafetch; w3lib; Chromium headless; SQLite 3; textcat 1.0-6 (R); AI4Privacy model accessed via Hugging Face Transformers. The spider and pipeline configuration files are available in the code repository at https://github.com/maciek85k/scraper.

### Method validation

We validate our web-scraped StepStone dataset against Lightcast as a commercial service. To maximise the reliability of this comparison, we deliberately selected a reporting window that begins one month earlier and ends one month later than our scraping period, thereby aligning with StepStone's one-month OJA posting schedule. For the overlapping period (April–July 2023), Table 3 presents Lightcast's observations, while Table 4 displays the corresponding data collected by our crawler. These tables summarise the number of OJAs and highlight the overlap between the sources. Overlap was identified via exact URL matching, providing a precise and unambiguous link between the two datasets that avoids the noise introduced by text-based deduplication.

**Table 3:** Lightcast data and overlap with scraped data.

	Lightcast	Overlap with Scrape (%)
Total	5,325,341	2091 (0.04)
AI OJA	45,703	32 (0.07)
Distinct Employer	404,514	849 (0.21)

**Table 4:** Scraped Data and Overlap with Lightcast Data.

	StepStone	Overlap with Lightcast (%)
Total	342,452	2091 (0.61)
AI OJA	3380	32 (0.92)
Distinct Employer	42,350	849 (2.04)

### Overlap statistics

Table 3, Table 4 present the overlap between the scraped StepStone dataset and the Lightcast corpus for the same collection window (April–July 2023), broken down by total OJAs, AI-related OJAs (identified via the OECD (2023) keyword list [15] in our data and an equivalent keyword filter in Lightcast), and distinct employers.

### Interpreting the URL-level overlap

The URL-level overlap reported in Table 3, Table 4 is low (0.61% of scraped postings; 0.04% of Lightcast records) and should be read as a strict lower bound on the true vacancy-level overlap rather than as a measure of it. Three mechanisms jointly account for the low rate. First, Lightcast aggregates postings from thousands of sources, including employer career sites, applicant-tracking systems, and multiple job boards, and deduplicates across these sources, retaining a single canonical record per vacancy. When a vacancy appears both on StepStone and, for example, on the employer’s own career site, the URL retained in Lightcast’s record typically points to a source other than StepStone. Exact URL matching therefore detects only the subset of jointly covered vacancies for which the StepStone URL happens to be the record Lightcast retained. Second, the same StepStone posting can be reachable under several URL variants (e.g., carrying different tracking parameters), so even postings that both systems collected from StepStone itself may fail an exact string match. This is common: 75.5% of the Lightcast records pointing to StepStone carried a redirect-wrapped or parameterised URL and could be compared only after normalisation, and variants beyond this pattern, such as a new posting ID after re-posting, still prevent an exact match. Third, the two systems crawl on different schedules within a posting’s roughly one-month lifetime, so short-lived postings may be captured by one crawler only. To gauge how much larger the true overlap is, we additionally compared the two sources at the text level. For the 18,607 Lightcast records that point to a StepStone URL, we matched the URL slug, which encodes the job title, the employer, and the location, while ignoring the numeric posting ID that changes when an advertisement is re-posted. Under this text-based criterion, 73.5% of these records (13,671) have a matching posting in our scrape, compared with 11.6% under exact URL matching. The low URL-level overlap is thus the expected outcome of cross-source deduplication and URL instability rather than evidence that the two datasets cover disjoint vacancy populations; the distributional comparison presented below therefore provides the more informative validation of coverage.

### Daily distribution of collected OJAs

Fig. 1 illustrates the intersection of the two data sources. This intersection comprises the 2091 postings appearing in both Lightcast and our own scrape. The left side of the figure shows how the Lightcast collection is distributed over time, while the right side provides a description of our scraper. Note that the right panel includes duplicate records — the same posting may be retrieved across multiple crawl sessions while it remains live on StepStone. The pipeline collected over 800,000 raw OJA records in total; after deduplication, 342,452 unique postings were retained. Lightcast is represented in blue, while our scrape is in orange. Although both datasets contain overlapping data, the percentage of the overlap is rather small, consistent with the mechanisms discussed above: cross-source deduplication on Lightcast’s side, URL variants of the same posting, and differing crawl schedules.

### Regional coverage

To understand the validity of our dataset's regional information, we aggregated cities on a NUTS-2 level using a conservative string-matching routine: city names extracted from the postings were compared, after trimming whitespace, against the official list of German municipalities and their NUTS-2 codes, and only exact matches were counted. Postings whose city name did not match exactly (for example due to misspellings or district-level names) were treated as lacking regional information. This conservative approach maximises accuracy, so underestimation is more likely than overestimation. The Lightcast data provides regional location information at this level as well. Table 5 shows that 80.31% of Lightcast OJAs mention at least one region, while our self-scraped dataset achieves a very similar 79.18% coverage at a difference of just over one percentage point, confirming that the single-portal pipeline captures the geographic footprint of the German labour market almost as broadly as a multi-portal commercial aggregator. This finding is consistent with Napierala et al. [16], who show that high-skilled occupations and more industrialised regions are systematically over-represented in OJA data across EU Member States.

**Table 5:** Regional coverage at NUTS-2 level.

	OJAs with city information	Coverage %
Lightcast	3,886,164	80.31
StepStone	272,685	79.18

### Spatial distribution of OJAs and AI-related OJAs

**Fig. 2:**
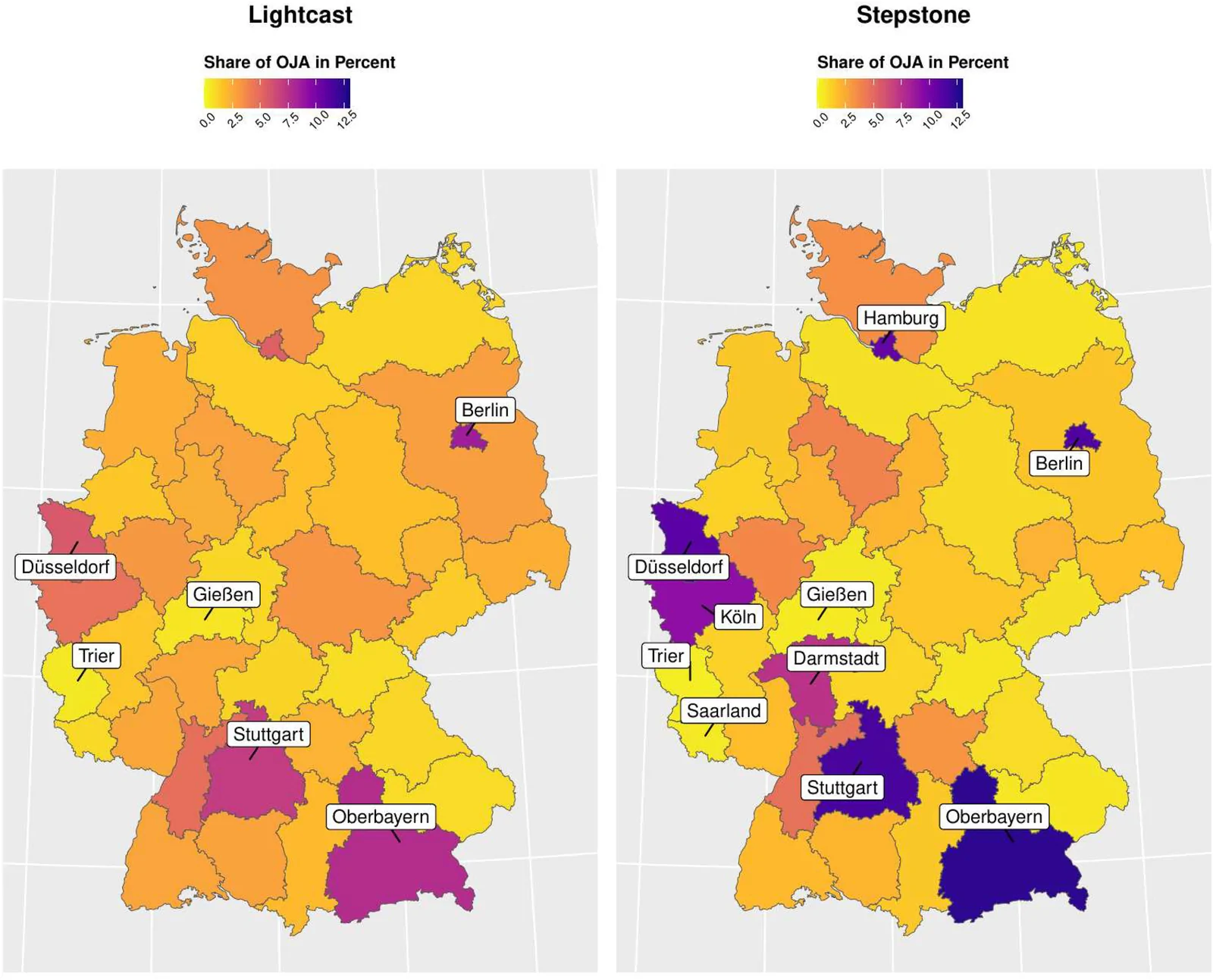
Share of OJA per NUTS-2 region (Lightcast vs. StepStone scrape).

Fig. 2 shows the share of all OJAs per NUTS-2 region for both datasets. The spatial patterns are broadly consistent: Berlin, Oberbayern (Munich metropolitan area), and Stuttgart hold the highest shares in both sources. In the Lightcast dataset, Berlin holds 8.5% of all OJAs, Oberbayern 7.7%, and Stuttgart 7%. In our self-scraped dataset, Berlin holds 12% of the OJAs a higher concentration reflecting StepStone's focus on professional occupations in major urban centres. At the lower end of the distribution, regions contribute around 0.5% of all OJAs in both sources (e.g., Oberfranken, Gießen, Trier).

### Distributional similarity

To quantify the visual similarity in Fig. 2 beyond a descriptive comparison, we treat the regional shares of each source as discrete probability distributions over the 38 German NUTS-2 regions and compare them directly. The Kullback–Leibler divergence of the StepStone share distribution from the Lightcast distribution is Dₖₗ = 0.096 nats (symmetric Jensen–Shannon divergence: 0.024), indicating a small departure from the commercial benchmark. The rank ordering of regions is likewise preserved: the Spearman rank correlation between the two share vectors is ρ = 0.91 (Pearson r = 0.93; both p < 0.001). These statistics support the claim that, despite the low URL-level overlap, the single-portal scrape reproduces the regional structure of the German OJA landscape captured by Lightcast.

**Fig. 3:**
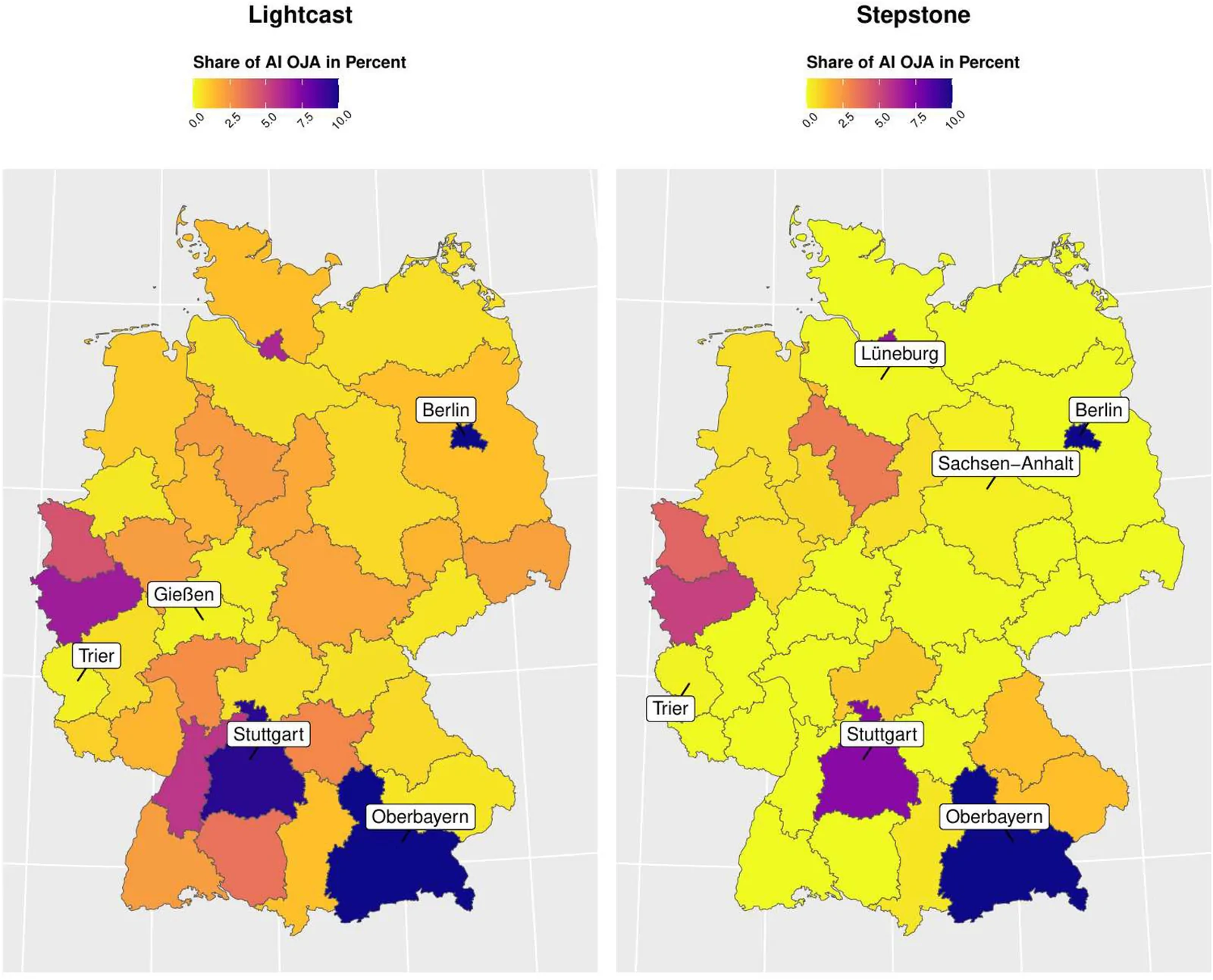
Share of AI-related OJA per NUTS-2 region (Lightcast vs. StepStone scrape).

Fig. 3 replicates the analysis for AI-related OJAs, defined as above using the OECD (2023) keyword list [15]. Lightcast categorises only a small fraction of postings as AI-related: 0.86% of the 5,325,341 OJAs contain at least one AI-related keyword. Using a simple and conservative string-match approach, we identified 3380 postings that mention AI in our self-scraped data (0.98% of all scraped postings). We excluded string combinations with special characters (e.g., C++) because regular expressions produce errors with such characters. Berlin, Oberbayern, and Stuttgart remain the dominant regions in both datasets, but the two sources diverge on which regions rank lowest: Lightcast highlights Gießen and Trier, while the scraped data shows lower AI shares in Sachsen-Anhalt and Lüneburg. This divergence reflects the single-portal limitation: analysing only a niche subset of OJAs amplifies geographic sampling variance. Researchers focusing on AI skill demand in less populated regions should therefore cross-validate with a broader data source.

## Limitations

1. Single-portal coverage. Only StepStone is crawled, which means that niches or sectors where StepStone has lower market penetration — including hospitality, health and social care, and public administration — are under-represented relative to the German job market as a whole [9]. This is consistent with our validation finding that the scraped data performs well for knowledge-intensive and digitally-affine occupations but should be used cautiously for blue-collar or public-sector analyses. Researchers requiring comprehensive coverage should complement the scraped data with other portals or with a commercial provider [7].
2. Geographic bias. Regions with larger populations are over-represented in our sample relative to the Lightcast benchmark [16,17]. Low-population regions may yield few or no AI-related OJAs in the scraped dataset. Single-portal approaches should therefore be used with caution for fine-grained regional analyses of niche occupational categories.
3. Keyword-based AI classification. The OECD AI skill keyword list used to identify AI-related OJAs excludes search terms containing special characters (e.g., C++), because regular expressions produce errors with such strings. This may cause a marginal undercounting of AI-related postings in technical fields.
4. Temporal scope. The pipeline was run for four months (April–July 2023). This window may not capture seasonal variation in labour demand. Researchers requiring longitudinal data should schedule repeated runs and implement a versioning strategy for the output files.
5. Scraper fragility. StepStone frequently updates its front-end layout and CSS class names. The CSS selectors used for data extraction may break after a platform update, requiring manual inspection and patching of the spider before the next run. The specific selectors used during the April–July 2023 collection are listed in Table 6.

**Table 6:** CSS extraction selectors used during the April–July 2023 collection. several class names are auto-generated hashes (e.g. resultlist-10m88ja) that StepStone regenerates on each front-end rebuild, so selectors must be re-checked before each run.

Field	CSS selector
job-posting links	a.resultlist-10m88ja
title	title
date_post	span.sc-fzoLsD.fYZyZu
firm_id	a.listing-content-provider-1u9ftq4::attr(href)
firm	a.listing-content-provider-1u9ftq4::text
about_work	span.listing-content-provider-1u79rpn
info_1	span.listing-content-provider-pz58b2:first-child
6. Legal environment. The data were collected without StepStone’s explicit permission: we contacted the platform repeatedly but received no response. Although the pipeline accessed only publicly available pages, obeyed robots.txt, and throttled its request rate, StepStone’s Terms of Service may be read to prohibit automated collection, so contractual claims or claims under the sui generis database right (Directive 96/9/EC) cannot be fully excluded. The text-and-data-mining exception for scientific research (Art. 3 Directive (EU) 2019/790; § 60d UrhG) provides an affirmative legal basis. The wider landscape is evolving: Art. 40(12) of the Digital Services Act (Regulation (EU) 2022/2065) grants researchers a right to collect publicly accessible data from designated very large online platforms, and the EU AI Act (Regulation (EU) 2024/1689) imposes transparency obligations should the released corpus be reused as training data. Researchers adapting this pipeline should reassess this framework at the time of collection.

## Ethics statements

The data were collected from publicly accessible employer-generated job advertisements on the StepStone job portal. No personal data pertaining to individual job applicants or users were intentionally collected. Contact information inadvertently embedded in posting text (e.g., recruiter email addresses or phone numbers) was detected and redacted using a locally deployed large language model (AI4Privacy) prior to data release, and no data were transmitted to external servers during this step. The pipeline respects StepStone's robots.txt directives. Data collection and release comply with GDPR (Regulation (EU) 2016/679) and the German Federal Data Protection Act (BDSG). Institutional ethics review was not required for this study, as data were collected solely from publicly accessible, employer-generated content and no personal data on individuals were intentionally processed.

Supplementary material and/or additional information [OPTIONAL]

## Related research article

None.

## CRediT author statement

Matthias Kapa: Data curation, Formal analysis, Investigation, Methodology, Software, Validation, Visualization, Writing – original draft, Writing – review and editing.

Kerstin J. Schaefer: Conceptualization, Funding acquisition, Project administration, Resources, Supervision, Writing – review and editing.

## Declaration of competing interests

The authors declare that they have no known competing financial interests or personal relationships that could have appeared to influence the work reported in this paper.

## Data Availability

I have shared the link to the data in my manuscript.
